# Bacterial Stress and Mortality may be a Source of Cell-free Enzymatic Activity in the Marine Environment

**DOI:** 10.1264/jsme2.ME18123

**Published:** 2019-02-23

**Authors:** Federico Baltar, Daniele De Corte, Taichi Yokokawa

**Affiliations:** 1 Department of Limnology & Bio-Oceanography, Center of Functional Ecology, University of Vienna Althanstraße 14, 1090, Vienna Austria; 2 Department of Marine Science, University of Otago New Zealand; 3 NIWA/University of Otago Research Centre for Oceanography New Zealand; 4 Japanese Agency for Marine-Earth Science and Technology (JAMSTEC) Japan

**Keywords:** marine environment, extracellular enzymatic activity, cell-free enzymatic activity, bacterial mortality

## Abstract

Marine microbes play a central role in driving biogeochemical cycles. Microbial extracellular enzymatic activities (EEA) are the ‘gatekeeper’ of the marine carbon cycle, and these enzymes may be found attached to cells or dissolved (cell-free). Recent studies indicated that the proportion of dissolved enzymatic activity is generally similar to (if not higher than) cell-attached activity. Thus, it is critical to understand the sources and sinks of cell-free EEA in the ocean. We herein empirically tested whether bacterial stress and mortality (induced by mitomycin C) are a source of the cell-free EEA of alkaline phosphatase (APase), beta-glucosidase (BGase), and leucine aminopeptidase (LAPase). We found that bacterial stress and mortality caused relative increases in the proportion of dissolved relative to total EEA of up to 10.5% for APase, 13.5% for BGase, and 7.3% for LAPase. These relative increases in dissolved EEA corresponded to absolute increases in the cell-free pool of 4.8, 7.2, and 3.8% for APase, BGase and LAPase, respectively. Collectively, our results contribute relevant information on the origin of free dissolved extracellular enzymes in marine waters, indicating that bacterial stress and mortality are a source of cell-free enzymatic activity and suggesting a potential link between microbial interactions and the degradation of organic matter via the release of cell-free enzymes.

Microbes play a central role in driving marine biogeochemical cycles (5, 20). Marine pelagic microbes preferentially utilize high-molecular-weight dissolved organic compounds (12); however, most are too large to directly pass through the semi-permeable prokaryotic cell membrane (30). Microbes use extracellular enzymes to hydrolyze these compounds before incorporating them into prokaryotic cells, which places extracellular enzymatic activities (EEA) as the ‘gate-keeper’ of the C cycle (4), and also of the N and P cycles (due to the activities of protease and organic-P hydrolysis). Total EEA is the sum of cell-free (*i.e*., dissolved) enzymes, enzymes attached to or in close association with the cell, and those within the periplasmic space (22). Most EEA in the marine environment were previously anticipated to be associated with cells, with only marginal contributions from cell-free enzymes (15, 16, 22, 28). However, recent evidence indicates that the contribution of cell-free to total EEA may be similar to, if not higher than the cell-associated pool (3, 7–9, 19, 23, 26). The important role of cell-free EEA in the marine environment and its implications were recently discussed in a perspective article, raising awareness of the importance to study this ‘living dead’ pool of enzymes in order to fully constrain marine biogeochemical cycles (11). To obtain a more complete understanding of the role of cell-free EEA, it is imperative to understand the potential sources and controls of the activities of these cell-free enzymes.

Previous studies reported that enzymes may be actively released by bacteria in response to appropriate substrates (2), bacterial starvation (1), and changes in cell permeability (17). Furthermore, changes in temperature (9) and dissolved organic matter origins and/or compositions (*e.g*. cover of seagrass versus mangrove) were recently shown to affect the proportion of cell-free to total EEA (10). In addition, protist grazing on bacterial natural communities has been shown to affect the release of cell-free enzymes (13).

We herein investigated whether bacterial-induced stress and mortality are a source of cell-free EEA. We examined the effects of bacterial stress and mortality, induced by mitomycin-C (MITO-C), on the proportion of the cell-free relative to total EEA of a glycolytic enzyme (beta-glucosidase [BGase]), proteolytic enzyme (leucine aminopeptidase [LAPase]), and alkaline phosphatase (APase). MITO-C is an antibiotic that inhibits DNA replication and activates DNA repair mechanisms that induce lysogenic viruses to enter the lytic cycle (29). Thus, MITO-C was used herein to induce bacterial stress and simulate bacterial mortality, *e.g*. by viral mortality, similar to nature. We investigated whether MITO-C increases the relative proportion of dissolved to total EEA and/or the absolute cell-free pool. We hypothesized that an increase in bacterial stress and mortality may cause the liberation of bioactive enzymes into media, thereby increasing the relative and absolute cell-free EEA pools. The results obtained propose bacterial mortality-related stress as a source of cell-free enzymes in the marine environment.

## Materials and Methods

### Experimental set-up

**Fig. 1:**
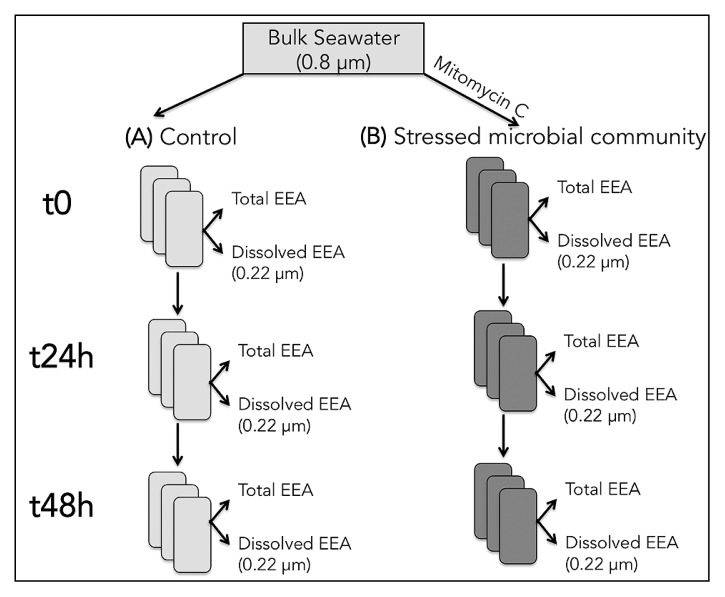
Diagram showing the experimental design. See the Materials and Methods section for detailed information.

Seawater was collected from Yokosuka Bay (Japan), and passed through a 0.8-μm polycarbonate filter (to remove grazers and big particles) at <100 mbar vacuum. This water was then used to prepare different treatments: (A) 0.8 μm-filtered seawater control, and (B) 0.8 μm-filtered seawater with stimulated bacterial mortality (using MITO-C, 0.9 μg mL^−1^ final concentration) (Fig. 1). The proportion of dissolved relative to total EEA in treatments A (Adiss) and B (Bdiss) was also analyzed as described below. All these treatments were prepared in triplicate 50-mL tubes, and incubated at an *in situ* temperature in the dark for up to 48 h. Subsamples for measurements were taken 1, 24, and 48 h after the initiation of the incubation. Besides EEA, changes in bacterial and viral abundance were quantified as described below.

### Bacterial and viral abundance

Flow cytometry after nucleic acid staining was used to enumerate bacteria and viruses (14, 18, 27). Briefly, 1.5-mL samples were fixed with glutaraldehyde (0.5% final concentration), held at 4°C for 10–30 min, shock-frozen in liquid N_2_, and kept at −80°C until analyzed.

Immediately prior to the flow cytometry analysis, samples were thawed to room temperature and 0.5-mL subsamples were stained with SYBR Green I (Molecular Probes) in the dark for 10 min. Prokaryotes were enumerated on an Attune flow cytometer (Thermo Fisher Scientific, Waltham, MA, USA) by their signature in a plot of green fluorescence *versus* side scatter (14, 18).

Viral abundance was also measured by flow cytometry after SYBR Green I staining (14). Prior to the analysis, samples were thawed and 0.5-mL subsamples were stained with SYBR Green I (Molecular Probes, Eugene, OR, USA) at a final concentration of 0.5× the manufacturer’s stock solution at 80°C for 10 min in the dark. Viruses were enumerated on the Attune flow cytometer as described above for prokaryotic abundance.

### EEA

The hydrolysis of the fluorogenic substrate analogues L-leucine-7-amido-4-methylcoumarin, 4-methylumbelliferyl (MUF)-β-D-glucoside, and MUF-phosphate was investigated to estimate the potential activity rates of LAPase, BGase, and APase, respectively (21). The same procedure as that previously described was followed (6–8). Briefly, EEA was assessed after substrate addition and an incubation using a spectrofluorometer with a microwell plate reader (Biotek Cytation 3 Imaging Multi-Mode Reader) at excitation and emission wavelengths of 365 and 445 nm, respectively. Samples (300 μL) were incubated in the dark at an *in situ* temperature for 1.5–3 h. An increase in fluorescence over time was transformed into hydrolytic activity using a standard curve established with different concentrations of the fluorochromes MUF and MCA added to 0.22 μm-filtered sample water. A final substrate concentration of 250 μmol L^−1^ was used to measure BGase, APase, and LAPase activities.

The total and dissolved fractions of EEA were distinguished as previously described (7–9). Bulk 0.8 μm-filtered seawater was used for total EEA. Regarding dissolved EEA, samples were gently filtered through a low protein-binding 0.22-μm Acrodisc Syringe filter (Pall) following a previously reported protocol (25). In the present study, dissolved (cell-free) EEA were defined as EEA recovered in the filtrate. Total and dissolved EEA were assessed on six technical replicate samples per treatment and sampling point.

The effects of bacterial mortality and cell-free EEA were calculated for both the ‘relative proportion of cell-free to total EEA’ and as the ‘absolute change in the cell-free pool’. At the same time, the ‘relative proportion of cell-free to total EEA’ was presented in terms of the % increase in the contribution of cell-free enzymes to total enzyme activity by bacterial mortality relative to the contribution of cell-free enzymes to total enzymes in the control (*i.e*., %Bdiss–%Adiss), where A refers to the control and B is the mitomycin-stressed treatment, and Adiss and Bdiss are the corresponding dissolved EEA fractions of each (*i.e*., % dissolved of A=[{100*Adiss}/A]: and % dissolved of B=[{100*Bdiss}/B]). However, this representation of subtracting %Adiss from %Bdiss does not reflect the reduction in (inhibition of) EEA (and BA) caused by MITO-C. Thus, this representation was corrected by calculating the contribution of Adiss to B and subtracting this contribution from %Bdiss (*i.e*., %Bdiss–[{Adiss/B}*100]) to provide a more realistic view of the response. Although this representation provides information on the relative change in cell-free relative to total EEA, it merely gives a partial view of the response because it is possible to detect an increase in % values even if there is no increase in the pool of cell-free enzymes by mortality. Therefore, since this representation of the response does not reflect the (absolute) change in the pool size of cell-free enzymes by mortality, we then calculated absolute changes in the pool of cell-free enzymes by subtracting Adiss from Bdiss and calculating the % of the difference increase in the activity of dissolved enzymes due to mitomycin bacterial mortality relative to Control A (*i.e*., [{Bdiss–Adiss}/A]*100).

### Statistical analysis

In all analyses, parametric assumptions were initially checked using the Shapiro–Wilk test for normality and Levene’s test for equal variance. Where appropriate, data were log-transformed to meet normality assumptions prior to analyses. Tukey’s HSD tests were performed to assess the significance of differences between treatments. All analyses were run using JMP^®^ 10.0.0 software (SAS Institute, Cary, NC, USA).

## Results and Discussion

**Fig. 2:**
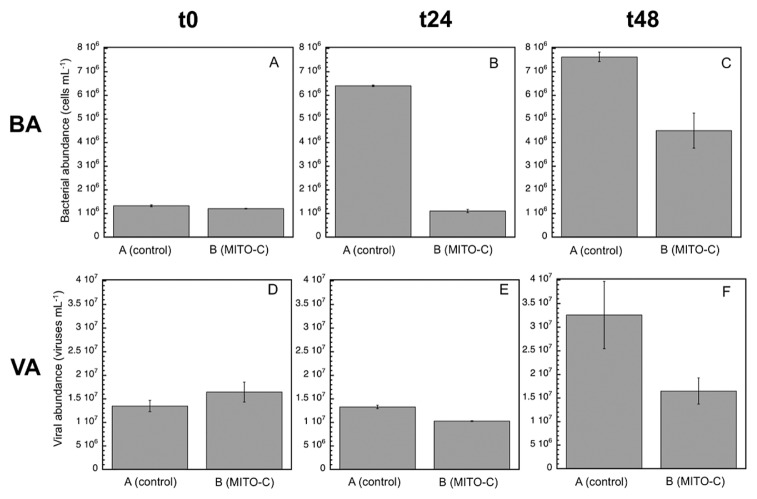
Mean (±SD, *n*=3) bacterial abundance (A, B, C) and viral abundance (D, E, F) at time zero (A, D), 24 h (B, E), and 48 h (C, F). Treatment A: control (0.8 μm-filtered seawater); Treatment B: MITO-C addition to 0.8 μm-filtered seawater. BA: Bacterial abundance; VA: Viral abundance. Note that time zero was approximately 1 h after the addition of MITO-C.

At time zero (approximately 1 h after the addition of MITO-C), abundance in the control (1.3×10^6^ cells mL^−1^) was similar to that with the MITO-C treatment (1.2×10^6^ cells mL^−1^) (Fig. 2A). Viral abundance followed a very similar pattern to bacterial abundance (Fig. 2A and D), showing similar numbers of viruses between the control and mitomycin treatment at time zero.

After 24 h, a significant (*P*<0.05, Tukey HSD) 5-fold increase in bacterial numbers was observed in the control, contrasting with an actual decrease of approximately 10% in bacterial numbers with the MITO-C treatment (indicating bacterial stress and mortality) (Fig. 2B). Despite the increase observed in bacterial abundance in the control from time zero to 24 h, viral numbers remained similar in the control and with the MITO-C treatment after 24 h (approximately 1 to 1.3×10^7^ viruses mL^−1^) (Fig. 2E). This caused a marked difference in the virus to bacteria ratio between the control and MITO-C treatment from approximately 2 to 10, respectively. Since the A (control) and B (0.8-μm filtered plus MITO-C) treatments were both filtered through a 0.8-μm filter, which generally stimulates the growth of bacterioplankton by removing most of the grazers, thereby reducing grazing pressure bacteria (as observed in A), the decrease observed in bacterial cell numbers in the MITO-C treatment during those 24 h indicated that bacterial stress and mortality were more important than growth (*i.e*., a compensation of bacterial growth by mortality) in the MITO-C treatment.

After 48 h, bacterial abundance remained at a similar value as that after 24 h (6–7×10^6^ cells mL^−1^) in the control, but had begun to increase (from 1.1×10^6^ to 4.6×10^6^ cells mL^−1^) in the MITO-C treatment (Fig. 2C). After 48 h, viral abundance significantly (*P*<0.05, Tukey’s HSD) increased to 1.6-fold that with the MITO-C treatment (from 1×10^7^ to 1.6×10^7^ viruses mL^−1^ at 24 and 48 h, respectively), and by 2.5-fold that of the control (from 1.3×10^7^ to 3.3×10^7^ viruses mL^−1^ at 24 and 48 h, respectively) (Fig. 2F). These results suggest that the growth of bacterial cells in the control was already reaching its maximum after 48 h and also that new bacterial growth was taking place with the MITO-C treatment after 48 h; once the effects of MITO-C had disappeared and/or once specific members of the community (more adapted to overcome the effects of MITO-C) grew to compensate for the mortality caused by MITO-C.

**Fig. 3:**
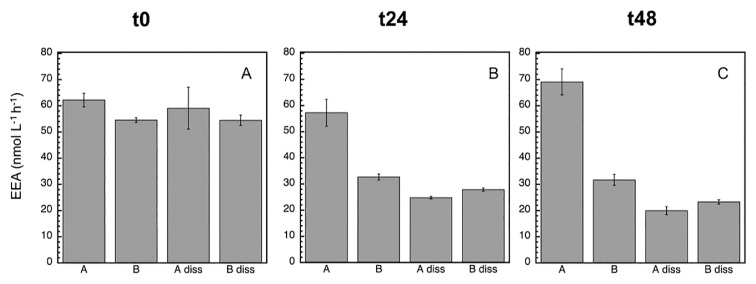
Mean (±SD, *n*=3) alkaline phosphatase (APase) activity at time zero (A), 24 h (B), and 48 h (C). In addition to the assessment of the total EEA of treatments A and B, the dissolved EEA of treatments A (‘diss A’) and B (‘diss B’) were analyzed. See the legend of Fig. 2 for a description of treatments A and B.

**Fig. 4:**
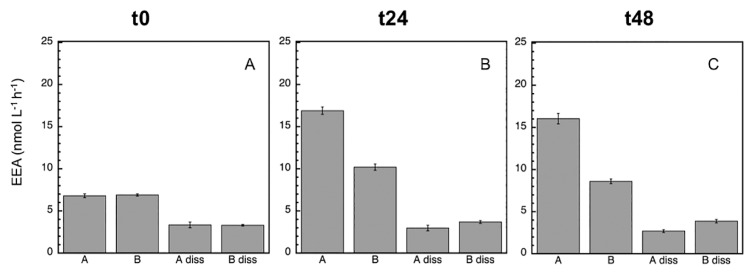
Mean (±SD, *n*=3) beta-glucosidase (BGase) activity at time zero (A), 24 h (B), and 48 h (C). In addition to the assessment of the total EEA of treatments A and B, the dissolved EEA of treatments A (‘diss A’) and B (‘diss B’) were analyzed. See the legend of Fig. 2 for a description of treatments A and B.

**Fig. 5:**
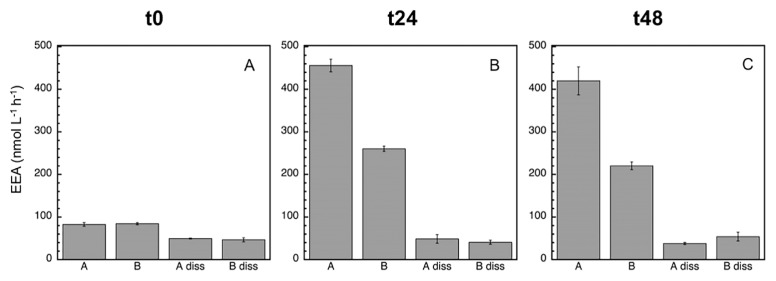
Mean (±SD, *n*=3) leucine aminopeptidase (LAPase) activity at time zero (A), 24 h (B), and 48 h (C). In addition to the assessment of the total EEA of treatments A and B, the dissolved EEA of treatments A (‘diss A’) and B (‘diss B’) were analyzed. See the legend of Fig. 2 for a description of treatments A and B.

Since bacterial growth inhibition and mortality were detected with the MITO-C treatment (particularly in the first 24 h), the next step was to analyze potential EEA responses. The most active EEA was LAPase, followed by APase and BGase (Fig. 3, 4, and 5), which is a common pattern generally found in marine environments (22). At time zero, the proportion of dissolved (cell-free) relative to total EEA in the control was higher for APase (95%) than for BGase (49.2%) and LAPase (60.2%) (Table 1). These proportions of dissolved relative to total EEA are consistent with previous findings on a wide variety of marine environments, suggesting the paramount role of cell-free EEA in the degradation of organic matter in the marine environment (3, 7–10, 19, 23, 24, 26). At the initial time, a marked inhibitory effect due to mitomycin was detected for APase (12.3%), but not for BGase or LAPase (Fig. 3 and Table 1). However, no significant differences were noted between the dissA and dissB treatments for any of the enzymes (*P*>0.05, Tukey’s HSD), suggesting that although there was a change in the relative contribution of dissolved to total EEA, the absolute change in the pool size of cell-free enzymes induced by mortality was not significant.

After 24 h, the inhibitory effect caused by MITO-C on EEA was more pronounced (*ca*. 40%), and clearly detected for all enzymes (Fig. 3, 4, 5, and Table 2). The strong inhibition of EEA caused by MITO-C at 24 h coincided with decreases in bacterial growth and abundance. Consistent with the stronger inhibition of EEA in response to MITO-C at 24 h, a more pronounced increase in the proportion of cell-free relative to total EEA was observed (Fig. 3, 4, 5, and Table 2). Significant changes (*P*<0.05, Tukey’s HSD) in cell-free EEA (*i.e*., Bdiss–Adiss) were observed for APase (Fig. 3B) and BGase (Fig. 4B) (but not for LAPase [Fig. 5B]). Relative increases in the % of cell-free relative to total EEA of 42 and 18.7% were found for APase and BGase, respectively (Table 2). When these relative increases were corrected by taking into account the original pool of cell-free EEA and percentage of MITO-C inhibition (see the Materials and Methods section for details), actual relative increases in cell-free to total EEA translated into 9.4% for APase and 7.1% for BGase. These relative increases in dissolved EEA corresponded to absolute increases (*i.e*., relative to actual EEA in control A) of approximately 5.4 and 4.3% of total APase and BGase activities.

After 48 h, the inhibitory effect caused by MITO-C on EEA was detected for all enzymes, and increased by approximately 6–14% from the 24-h time point (*i.e*., from *ca*. 40% at 24 h to 46–54% at 48 h) (Fig. 3, 4, 5, and Table 2). Thus, although the inhibitory effect did not increase in the 24–48 h period as much as in the 0–24 h period, the cumulative inhibitory effect caused stronger inhibition to occur after 48 h. Consistent with this inhibition in response to MITO-C, a higher relative increase in the cell-free relative to total EEA (*i.e*., Bdiss–Adiss) was observed (Fig. 3, 4, 5, and Table 2). Significant changes (*P*<0.05, Tukey’s HSD) in cell-free EEA were found for all three enzymes (Fig. 3C, 4C, and 5C). Relative increases in the % of cell-free relative to total EEA of 44.6, 28.1, and 15.5% were found for APase, BGase, and LAPase, respectively (Table 2). Taking into account the original pool of cell-free EEA and the percentage of MITO-C inhibition, actual relative increases in cell-free to total EEA were 10.5% for APase, 13.5% for BGase, and 7.3% for LAPase. These increases corresponded to absolute increases of approximately 4.8, 7.2, and 3.8% of total APase, BGase and LAPase activities, respectively.

The percentage of dissolved relative to total EEA in the control (*i.e*., Adiss) decreased for all enzymes from t0 to t48 (Tables 1 and 2). The % of dissolved EEA has been shown to decrease in marine bacterial inoculums that actively start growing when relieved of grazing pressure (by the filtration and exclusion of grazers) or in response to organic matter enrichment (10). These findings suggest that actively responding populations (mostly copiotrophs) to sudden positive changes in top-down (*i.e*., decrease in grazing pressure) or bottom-up (*i.e*., organic matter input) control are more likely to rely, at least initially, on cell-associated than cell-free enzymes.

During the incubation, the percentage of the dissolved EEA rate of the control (Adiss) decreased (to less than half) for APase, and not for the BGase or LAPase (Fig. 3, 4, and 5). Consistently, the total EEA (A) of APase remained relatively unchanged, but markedly increased for BGase and LAPase in response to the enhanced growth caused by grazing pressure relief in the control. The total EEA (A) of APase was approximately 61 nmol L^−1^ h^−1^ at t0 and 69 nmol L^−1^ h^−1^ at t48 (Fig. 3A and C); whereas LAPase increased from 80 to >400 nmol L^−1^ h^−1^ (Fig. 5A and C) and BGase more than doubled from 7 to 16 nmol L^−1^ h^−1^ (Fig. 4A and C) during the same incubation time. Once cell-free enzymes are released, they will start to decay, and the resulting cell-free EEA pool is the result of the balance between cell-free EEA being produced/actively liberated by cells minus the decay of cell-free EEA. Thus, the lack of a change in total APase in the control despite the increase in bacterial growth indicates that the loss of cell-free APase due to cell-free enzyme decay over time was not counterbalanced by the similar production/release of new cell-free EEA by growing cells of the control (A), resulting in a decrease in cell-free APase rates with time in the control.

**Fig. 6:**
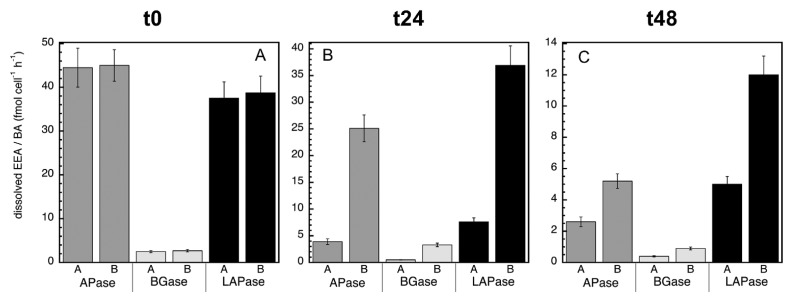
Normalized dissolved extracellular enzymatic activity by bacterial abundance (*i.e*., dissolved EEA: bacterial cell abundance) at time zero (A), 24 h (B), and 48 h (C) for APase (alkaline phosphatase), BGase (beta-glucosidase), and LAPase (leucine aminopeptidase). This ratio was calculated for the dissolved EEA of treatments A (‘A’) and B (‘B’). See the legend of Fig. 2 for a description of treatments A and B.

Overall, no significant differences were observed in the dissolved EEA:bacterial abundance ratio of the MITO-C treatments relative to the control for any of the enzymes at t0, (Fig. 6A). However, bacterial stress and/or induced mortality due to the addition of MITO-C increased the dissolved EEA:bacterial abundance ratio (at 24 and 48 h) over that of the control (Fig. 6B and C). This result also indicates that the bacterial species actively growing in the control (in response to the relief of grazing pressure due to the 0.8-μm filtration) produced low levels of cell-free enzymes. These results were consistent with the increase in cell-free EEA in response to the addition of MITO-C, as observed for APase, BGase, and LAPase. In conclusion, our results suggest that bacterial mortality is a substantial source of cell-free enzymatic activity. Based on these results, the contribution of cell-free enzyme activities due to cell stress and death appear to be relatively low (Table 2). However, our results represent only a fraction of cell-free enzymes that do not associate with particles, and, as such, is a conservative estimate since many enzymes after mortality may remain attached to (or adsorbed on) particle/cell fragments and may not pass through 0.22-μm filters (and may not appear in the dissolved fraction). Moreover, our results suggest that microbial interactions (*e.g*. via stress and mortality caused by antibiotic warfare or viral attacks) affect the cell-free EEA pool, indicating another link between microbial communities and organic matter degradation. Thus, since EEA is the rate limiting step in the degradation of organic matter, and cell-free EEA are a main component of total EEA, our results provide another crucial step towards understanding the forces that control energy flow in the ocean as well as the cycling of compounds that influence climate change, and may contribute towards building more accurate models of global biogeochemical cycles.

## Figures and Tables

**Table 1:** Initial (time zero) percentage of inhibition caused by mitomycin, percentage of dissolved extracellular enzymatic activity (EEA) in treatment A, and percentage of dissolved EEA in treatment B. APase: alkaline phosphatase, BGase: beta-glucosidase, LAPase: leucine aminopeptidase. See the Materials and Methods section for details on calculations.

	APase	BGase	LAPase
% mitomycin inhibited	12.3	−1.3	−2.2
% A diss	95.0	49.2	60.2
% B diss	99.9	48.0	55.4

**Table 2:** Percentage of inhibition caused by mitomycin, percentage of dissolved extracellular enzymatic activity (EEA) in treatment A, percentage of dissolved EEA in treatment B, relative percentage increase in dissolved relative to total EEA, corrected percentage increase in dissolved relative to total EEA, and absolute increase in the cell-free EEA pool after an incubation for 24 (t24) and 48 (t48) h. APase: alkaline phosphatase, BGase: beta-glucosidase, LAPase: leucine aminopeptidase. See the Materials and Methods section for details on calculations.

t24		**APase**	**BGase**	**LAPase**
	% mitomycin inhibited (=100–[100*B]/A)	42.9	39.6	42.9
	% dissolved of A (=[100*Adiss]/A)	43.3	17.6	10.7
	% dissolved of B (=[100*Bdiss]/B)	85.2	36.3	15.7
	% increase in dissolved EEA relative to total (=%Bdiss–%Adiss)	**42.0**	**18.7**	**5.0**
	% corrected increase in dissolved EEA relative to total (=%Bdiss–[{Adiss/B}*100])	**9.4**	**7.1**	−**3.1**
	% absolute increase in the dissolved EEA pool (=[{Bdiss–Adiss}/A]*100)	**5.4**	**4.3**	−**1.7**

t48	% mitomycin inhibited (=100–[100*B]/A)	54.1	46.4	47.5
	% dissolved of A (=[100*Adiss]/A)	28.9	16.9	9.1
	% dissolved of B (=[100*Bdiss]/B)	73.5	45.0	24.6
	% increase in dissolved EEA relative to total (=%Bdiss–%Adiss)	**44.6**	**28.1**	**15.5**
	% corrected increase in dissolved EEA relative to total (=%Bdiss–[{Adiss/B}*100])	**10.5**	**13.5**	**7.3**
	% absolute increase in the dissolved EEA pool (=[{Bdiss–Adiss}/A]*100)	**4.8**	**7.2**	**3.8**
